# The differential illusion memory for high-associated abstract concepts (DIM-HA) effect

**DOI:** 10.1007/s10339-024-01220-1

**Published:** 2024-08-17

**Authors:** Alejandro Marín-Gutiérrez, Emiliano Díez Villoria, Ana María González Martín

**Affiliations:** 1 Facultad de Educación y Psicología, Universidad del Atlántico Medio, Carretera de Quilmes, 37, Gran Canaria, 35017 Tafira Baja, Spain; 2 Departamento de Psicología Básica, Psicobiología y Metodología de las Ciencias del Comportamiento. Facultad de Psicología, Universidad de Salamanca, Salamanca, Spain; 3 Instituto Universitario de Integración en la Comunidad (INICO), Universidad de Salamanca, Salamanca, Spain

**Keywords:** Deese–Roediger–McDermott (DRM), Concreteness effect, Qualitative difference relationship theory, Associative activation theory, DIM-HA effect

## Abstract

A vast body of evidence has shown that concrete concepts are processed faster and more accurately than abstract concepts in a variety of cognitive tasks. This phenomenon is widely known as the concreteness effect, and explanations for its occurrence seem to reflect differences in processing and organization for both types of representations. While there is considerable evidence to support this concreteness effect, the nature of these differences is still controversial. In developing an explanation, we have proposed a relatively different approach from a false memory perspective using the Deese–Roediger–McDermott paradigm. To explore the implications of the association in creating false memories, we explore behavioral and electrophysiologically the false memory effect, where targets were manipulated according to their association strength and their concreteness. Results showed that false recognition rates differed significantly between concrete and abstract critical words when they were associated strongly with their respective lists, which led to a higher proportion of abstract false alarms both in behavioral and electrophysiological experiments. The principal outcome, which was called the DIM-HA effect, was discussed in terms of theories of associative activation and qualitatively different representation.

## Introduction

Concepts that allude to physical objects (such as puppy) are considered concrete, whereas concepts that refer to non-physical entities (such as idea) are considered abstract. Between these two categories, there is a continuum whereby all conceptual information is placed according to the links that the concepts share with the physical entities. In this context, the *concreteness effect* is defined as the processing advantage, in terms of speed and accuracy, for concrete over abstract concepts. Such concrete over abstract processing advantages has been demonstrated repeatedly from behavioral tasks (Bleasdale 1987; Crutch et al. 2009; Duñabeitia et al. 2009a, b; Schwanenflugel et al 1992), neuroimaging techniques (van Schie et al. 2005; Wang et al. 2010), and neuropsychological patients (Breedin et al. 1994; Crutch et al. 2009; Crutch and Warrington 2005, 2006). Despite robust observation of the effect, no convincing explanation has been provided unequivocally to account for its functional properties or its structural characteristics. That is, the factors that determines the concreteness effect are still a matter of controversy.

Paivio and Csapo proposed one of the first explanations for the concreteness effect in their *Dual Coding Theory* (Paivio and Csapo 1969). The authors hypothesize that the advantage of processing concrete over abstract terms is that concrete concepts are coded by both a verbal and an image system, whereas abstract concepts are coded only by the verbal system. Thus, concrete words are processed via two pathways, providing them with a higher imageability (Boles 1983; Richardson 1976).

Inspired by this dual coding logic, Schwanenflugel et al. (1992) discussed three different hypotheses that might explain the concreteness effect from a psycholinguistic point of view. The first one, known as the *Automatic-Imagery hypothesis*, states that concrete concepts, unlike abstract ones, are related to sensory experiences, which automatically makes them easier to remember as they are represented in two ways: verbally and imaginably. The second one is the *Strategic-Imagery hypothesis*, which claims that imagery is used deliberately when it is considered a helpful strategy. The final hypothesis is the *Context-Availability hypothesis,* which proposes that prior knowledge sets a frame in the processing of concrete and abstract concepts. Concrete concepts are supposed to be easier comprehended and recalled because related contextual knowledge is more accessible (e.g., the words have a higher context-availability). As the authors suggested, their results seem to support more the *Strategic-Imagery* hypothesis, since they had found the concreteness effects in the context-availability-controlled condition, following the imagery-rating and the directed intentional-memory tasks, but not after the context-availability-rating task. They also found that subjects reported the strategies that they used to encode the list; an imagery strategy showed concreteness effects for words controlled for rated context availability, but those not reporting it did not.

A different finding came from Kousta et al. (2011). The authors found that when controlling for imageability and context availability, there is a processing advantage for abstract but not for concrete words. Further experiments showed that this abstractness effect must have been produced due to the affective associations of the words, as abstract words tend to have higher emotional valence scores than concrete words.

To add more evidence for explaining the concreteness effect, Crutch and Warrington (2005) investigated the comprehension of abstract and concrete words from a neuropsychological approach. They conducted different word-matching tasks with a globally aphasic patient. Based on the results, they proposed the existence of qualitative differences in the representation of abstract and concrete concepts. According to them, concrete words are primarily organized based on taxonomic similarity, whereas abstract concepts seem to rely on the associative connections among them. As concreteness is considered a continuum, a concept can have both associative and categorical connections in different proportions. An associative network might provide the flexibility that is necessary to involve all the different, context-dependent meanings that abstract concepts need to be successfully processed. Thus, this established the birth of the *Qualitatively different representational* theory (QDR). This was the the first proof that the meanings of words, whether abstract or concrete, are grounded in representational systems with qualitatively distinct characteristics. More precisely, they demonstrated that an associative neural network represents abstract concepts, but not actual concepts. Additionally, it was discovered that their patient had noticeably more trouble recognizing high frequency abstract phrases than low frequency ones.

Further evidence for this QDR framework was found in a study with healthy participants using the visual-word paradigm (Duñabeitia et al. 2009a, b). After hearing an abstract word, the participants' attention tended to be captured more and earlier by a picture of a semantically associated object (among distractors) than after hearing a concrete word. Their results were interpreted as supporting evidence for the different organizational principles described by the QDR framework. Similarly, Crutch et al. (2009) also tested healthy participants who performed various odd-one-out tasks. They tended to process the connections between concrete words faster when they were similar than when they were associated. The reverse pattern was shown with abstract words.

Additionally, Mkrtychian et al. (2021) reported differences in behavioral and neurophysiological processing between concrete and abstract words that appear immediately after controlled acquisition, confirming that distinct neurocognitive mechanisms underlie these semantic representations. Specifically, in their distributed source analysis, they had indicated that bilateral temporo-parietal activation underpinning newly established memory traces, suggesting a crucial role of Wernicke’s area and its right-hemispheric homologue in both concrete and abstract word acquisition. However, in their EEG analysis, they found differences between novel words and control untrained pseudowords for concrete (~ 150 ms) and for abstract (~ 200 ms) words, which could mean that this effect may be explained in terms of processing rather than localization.

The analysis of the reviewed literature highlights the importance of the association strength, especially for processing abstract concepts. To further explore this assumption, the research we propose is aimed at contributing to this debate by using the Deese–Roediger–McDermott (DRM) paradigm (Deese 1959; Roediger and McDermott 1995), this is, from the false memory perspective. Although there has been evidence of the concreteness effect from the memory perspective, the analysis of memory disruption in healthy people should represent a convenient field of study to provide convergent evidence in this matter.

## The DRM paradigm

The DRM paradigm is based on the presentation of word lists to participants, each of which contains a keyword (or critical lure) that is intentionally suppressed during the study phase. Later, during the test phase, the researcher presents the lure, thereby activating the false memory effect. In other words, when participants are asked to recall/recognize the presented items, they frequently include the critical word. In the following paragraphs, we will discuss various theories explaining the false memories in the DRM paradigm.

## The associative-activation theory

In Gallo’s review (2006, 2010), the associative-activation theory describes that when participants study the list of words (e.g., *puppy, bark, pet, cat, friend*), they activate not only the word’s representation but also that activation spreads throughout associated concepts. At the test, the pre-activated representations (such as the critical word *“dog”*) are more available and more prone to be selected as part of the studied material (e.g., producing a false memory). This theory was part of the descriptions of the Activation/Monitoring Framework (e.g., Roediger et al. 2001).

This theory relies on the concept of spreading activation: when a word is studied, its representation within the conceptual system is activated, and this activation spreads out towards the representation of associated concepts that are placed close in the semantic network. To produce memory illusions, this theory proposes that participants may mistakenly believe that a non-presented associate occurred in the list because the lure items have more activation, being this a criterion of selection. In other words, the more activation shows a concept, the more familiar it sounds.

## Fuzzy trace theory

Fuzzy Trace Theory (Brainerd and Reyna 2002; Brainerd et al. 2008; Carneiro et al. 2012; Reyna and Brainerd 1995; Reyna and Brainerd 1998) is a psychological framework that explains how we form memories and how we retrieve them. Although this theory does not focus specifically on false memories, it can provide insight into how they can occur. According to Fuzzy Trace Theory, when we encode and store information, we produce both verbatim traces (exact details) and gist traces (meaning or essence). During memory retrieval, reliance on essence traces rather than verbatim traces can lead to the formation of false memories.

The *Fuzzy Trace Theory* explains false memories as follows:

*Gist-Based Encoding:* during memory encoding, people tend to derive the gist or meaning of an event rather than meticulously storing every detail. Specific details may be forgotten or altered because of this reliance on gist processing, resulting in memory distortions. False memories can occur when the essence of an event is remembered correctly, but the specific details are incorrectly reconstructed or influenced by external information. When recalling memories, people frequently rely on essence traces rather than exact verbatim traces. This process entails the reconstruction of memories using general knowledge and schema. Individuals with false memories may unconsciously fill in missing details with plausible or suggested information, leading them to believe that the reconstructed memory is authentic.

The *Fuzzy trace theory* also highlights the importance of suggestibility and misinformation in the formation of false memories. The reconstruction process can be influenced by external information, such as leading inquiries or misleading suggestions. If erroneous information corresponds to the essence or general significance of an event, it may be stored in memory, resulting in a false recall. The reliance on paraphrase processing during memory encoding and retrieval, according to the *Fuzzy Trace Theory*, may result in the formation of false memories. The reconstruction of memories based on general meaning, suggestibility, and misinformation can contribute to the formation of deceptive memories. False memories are complex phenomena influenced by a variety of cognitive and social factors, and the *Fuzzy Trace Theory* provides a framework for comprehending some of these processes.

To clarify the gap existing in the literature about the relationship between false memories and concreteness, the existing evidence parts from 2 studies, were they try to identify the factors but not directly. Thus, the question of whether there are differences between false memories of concrete and abstract words, is still unanswered.

The first study is the Pérez-Mata et al. (2002) one, where they used the DRM paradigm to compare false memory rates of concrete and abstract words. They found that concrete items were recalled correctly more often than abstract items and that false recall of non-presented words was higher for abstract lists than for concrete lists.

Another study, where concreteness was addressed (although not directly), was Roediger et al.’s (2001). However, the authors didn’t obtain a significant effect of concreteness on false memory. In this study, the authors mentioned that the most relevant factor in determining the production of false memory was association strength. This result is interesting because if we delve deeper into the concreteness effect theories, they affirm that association has a special and differential role in the processing of concrete vs. abstract concepts. Specifically, they mentioned the *Backward association strength* (BAS) of the lists which had already been reported by Deese (1959). BAS describes the “average tendency for words in the study list to elicit the critical item on a free association test” (Roediger et al. 2001). Therefore, lists with high BAS correlate with higher rates of false recall (Deese 1959; Roediger et al. 2001), as they probably promote activation of the critical concept more than those lists with low BAS.

## Concreteness and EEG

Combining the concepts of concreteness with EEG/ERP methodologies has provided nuanced insights into false memories. A study by West and Holcomb (2000) examined the ERP correlates of concrete and abstract words in false memory paradigms. Their findings revealed that concrete words produced larger N400 and LPC amplitudes compared to abstract words, indicating stronger semantic and retrieval processing for concrete words.

Moreover, research by Gutchess and Schacter (2012) highlighted that concrete words not only enhance true memory but also increase the susceptibility to false memories. Their ERP data showed that the neural signatures of false memories for concrete words closely mirrored those of true memories, emphasizing the role of sensory-rich encoding in the formation of vivid but erroneous recollections. Regarding the N400 component, Rabovsky et al. (2018) claimed that due to its ability to give an online measure of the brain's meaning processing, the N400 component of the event-related brain potential has generated a great deal of interest. But the fundamental mechanism is still poorly understood and hotly contested. We now offer a computationally clear explanation of this procedure and the developing sentence meaning representation. In a neural network model of sentence comprehension, they simulate N400 amplitudes as the change induced by an incoming stimulus in an implicit and probabilistic representation of meaning captured by the hidden unit activation pattern. They suggested that the mechanism underlying the N400 also drives implicit learning in the network. Our study can be useful to add convergence evidence in this N400 clarification of its functioning.

## The current study

In the context of the literature reviewed, the main goal of the present study was to explore the differences in false recognition of critical abstract words and critical concrete words, while determining the possible differential role of association strength. We manipulated the BAS, since this relationship effect is more important in the false memory parading than the FAS (Forward association strength). Thus, as a matter of control, we decided to use only the BAS to stain in line with the existent literature. We hypothesized that false recognition rates would vary in the different levels of concreteness and BAS. Forward and backward association strengths are concepts related to the connections between stimuli and responses. Forward association strength is the likelihood that a given stimulus (e.g., a word or image) will evoke a specific response. It is often measured by how readily one can predict the response given the stimulus. Backward association strength, on the other hand, refers to the likelihood that the response will evoke the original stimulus, essentially measuring the predictability of the stimulus based on the response. These concepts are critical in understanding associative learning and memory retrieval processes (Mandler 2014; Nelson et al. 2004).

To have a broad approach to this phenomenon, we included both behavioral and electrophysiological measures to add convergence evidence. That is, while the behavioral data could show the effect, the electrophysiological experiments could shed light on the time curse of the effect, its distribution across the scalp, and the nature of the components. Specifically, we are interested in exploring the time-window associated with the processing of semantic information (N400), but from a different perspective: analyzing the encoding phase, rather than the test phase. Thus, our hypothesis is that N400 will show different amplitudes and latencies for abstract and concrete studied lists and, for word lists highly and lowly associated with the list lure.

## Experiment one

As mentioned in the introduction, the role of concreteness in false memory production is still a matter of controversy. Besides, the effect has been explored in recall, but not in recognition memory, which relies on different cognitive processes (Gillund and Shiffrin 1984). To explore the relationship between association strength (BAS) and concreteness over false recognition, we created word lists weakly of strongly associated both with a concrete or abstract critical lure. As it was established in the introduction section, we hypothesized that a concreteness effect would emerge, and association strength would modulate this effect differently for concrete and abstract lists.

## Method

### Participants

Twenty-four psychology students (23 females) of the University of Salamanca took part in the experiment in exchange for course credits. Their mean age was 20.54 years. In all the experiments of this research, participants were asked to sign a written consent form.

### Ethical clearance

All participants in all the experiments showed on this paper signed an inform consent before participating. Ethics approval was obtained for the data collection as it was part of the doctoral dissertation of the first author.

### Design and materials

A two-by-two within-participants design was used with concreteness (abstract vs. concrete) and associative strength (high BAS vs. low BAS) as independent variables. The proportion of correct and false recognition were the dependent variables.

The word lists were created around 32 critical words (16 abstract and 16 concrete; mean: 2.69 and 5.92; range: 1.38–3.38 and 5.03–6.78 respectively) that were taken from the Algarabel’s normative study (1996). Additionally, indexes of word frequency, orthographic neighborhood, familiarity, word length, and imageability were obtained for the 32 critical words. Subsequently, the 32 critical words were introduced into NIPE, a web tool from the University of Salamanca’s research team on memory and cognition where up to date it is possible to obtain association norms for 4.051 words (Díez et al. 2006). For every critical word, we obtained twelve backward associates to build the experimental and distractor material, according to Brandt (1991) methodology. Table 1 contains the psycholinguistic properties for each critical word, as well as the association strength average for each list.

**Table 1:** Psycholinguistic properties of the list and critical lures

(ABS-HI)	BAS	FRE	L	N	IMA	CON	FAM	AoA	(ABS-LOW)	BAS	FRE	L	N	IMA	CON	FAM	AoA
Mentira	0.27	38.57	7	0	3.74	2.54	5.95	4.5	Moda	0.09	22.14	4	18	4.92	3.16	5.36	7.86
Tranquilidad	0.13	25	12	0	4.72	2.99	5.8	6.72	Inteligencia	0.08	58.93	12	0	3.52	2.21	6	7.1
Pobreza	0.13	21.25	7	0	5.03	3.29	5.57	6.58	Odio	0.08	34.29	4	2	4.2	2.06	4.66	6.08
Tristeza	0.19	36.43	8	0	4.28	2.38	5.45	6	Idea	0.07	195.71	4	1	4.17	1.88	5.86	6.64
Error	0.17	56.61	5	1	4.2	3.04	5.66	5.68	Peligro	0.08	78.75	7	0	4.86	3.34	5.59	4.26
Suerte	0.19	102.32	6	2	3.82	2.2	6.15	6	Soledad	0.07	62.5	7	0	5.07	2.34	5.02	8.46
Orden	0.15	159.46	5	0	3.62	3.13	5.52	5.58	Horror	0.07	29.82	6	0	5.09	2.81	5.21	6.38
Religión	0.14	49.82	8	1	4.37	2.41	4.41	5.06	Atención	0.05	132.32	8	1	3.97	3.38	4.95	6.24
Total	0.17	61.18	7.25	0.50	4.22	2.75	5.56	5.77		0.07	76.81	6.50	2.75	4.48	2.65	5,33	6,63

(CON-HI)	BAS	FRE	L	N	IMA	CON	FAM	AoA	(CON-LOW)	BAS	FRE	L	N	IMA	CON	FAM	AoA
Pintura	0,14	39.64	7	2	6.25	5.37	4.41	4	Traje	0.08	42.14	5	1	5.32	6.35	5.52	4.6
Película	0.22	107.5	8	1	6.5	5.87	6.05	3.78	Jefe	0.08	105.36	4	1	4.82	5.35	4.82	6.6
Examen	0.17	25.36	6	0	5.66	5.55	6.43	6.46	Escuela	0.08	56.79	7	3	6.36	5.8	4.59	3.4
Leche	0.21	54.11	5	2	5.96	6.65	5.69	2.64	Tela	0.07	23.75	4	12	5.01	6.32	5.57	5.34
Cama	0.23	136.43	4	20	5	6.78	5.42	2.42	Empresa	0.06	115.89	7	2	5.71	5.03	5	8.96
Playa	0.23	41.96	5	5	6.29	6.56	6.18	3.2	Plata	0.07	47.68	5	6	6.16	6.32	4.04	6.76
Ruido	0.14	57.5	5	6	5.34	5.37	6.05	4.42	Suelo	0.07	148.04	5	7	5.26	6.08	5.81	3.14
Color	0.14	128.75	5	4	5.92	5.14	5.68	2.84	soldado	0.08	23.93	7	2	5.64	6.23	4.84	5.86
Total	0.19	73.91	5.63	5.00	5.87	5.91	5.74	3.72		0.07	70.45	5.50	4.25	5.54	5.94	5.02	5.58

*BAS* = Backward association strength; *FRE* = Lexical frequency; *L* = Length; *N* = Semantic neighbors; *IMA* = Imaginability; *FAM* = Familiarity; *AoA* = Age of acquisition; *ABS* = Abstract; *CON* = Concrete; *HI* = High association; *LOW* = Low association

To create the context of different BAS conditions, we manipulate this measure to build four different conditions, such as, (1) Abstract—high BAS (the critical word was abstract and the words of the lists were strongly associated with it); (2) Abstract low BAS (the critical word was abstract and the words of the lists were weakly associated with it; (3) Concrete high BAS (The critical word was concrete and the words of the lists were strongly associated with it; (4) Concrete low BAS (the critical word was concrete, and the words of the lists were weakly associated with it). The set of lists was counterbalanced using a *Latin-square* approach, which allowed each list to act as both a stud*y* and a distracted material.

### Procedure

Up to three participants sat together in one room in front of 17’’ CTR computers' monitors. They were positioned so that they could not see the other participants' screens. Headphones were provided as protection against potential noise. Stimuli were presented in black letters (font-size: 18) on a white background. Each participant studied 16-word lists and the presentation order of the lists was counterbalanced. The participants read specific instructions at the beginning of each phase. The study phase started with the word “list” and its corresponding number centered on the screen (i.e “List 1”, to make sure that the participants would focus on the stimuli material. This list numeration remained on the screen for 3000 ms and was followed by the twelve words from the respective list. Each word was shown individually for 2000 ms. After a break od 1000 ms, the name of the next list appeared, followed by its content. This procedure was repeated until all 16 lists were presented to the participant.

Immediately after the study phase, a distractor task was presented to the participant. This task consisted in deciding whether several mathematical equations were correct or false. The distractor task was presented for 120 s in all the cases. The participants received feedback after each decision and the time limit for each decision was set at 100 s.

Finally, the participants were asked to complete a test phase, where the participants had to recognize a pool of words as studied or not studied (new). The questionnaire contained a total of 96 words formed of 16 critical words, 32 studied words (words no. 2 and 7 of each studied list), 32 distractors (words no. 2 and 7 of each non-studied list), and 16 critical control words (critical words associated with the non-studied lists). Each of the 96 trials began with a fixation cross displayed for 500 ms Afterward, a word was presented on the center of the screen and the participant had to decide whether that word had been studied before or not by pressing the keys “A” (“No.”) or “L” (“Yes.”). The word remained visible for the participant until a response was given (i.e., it was self-paced). After the participants’ response, a new trial began. Response keys were counterbalanced across participants.

**Fig. 1:**
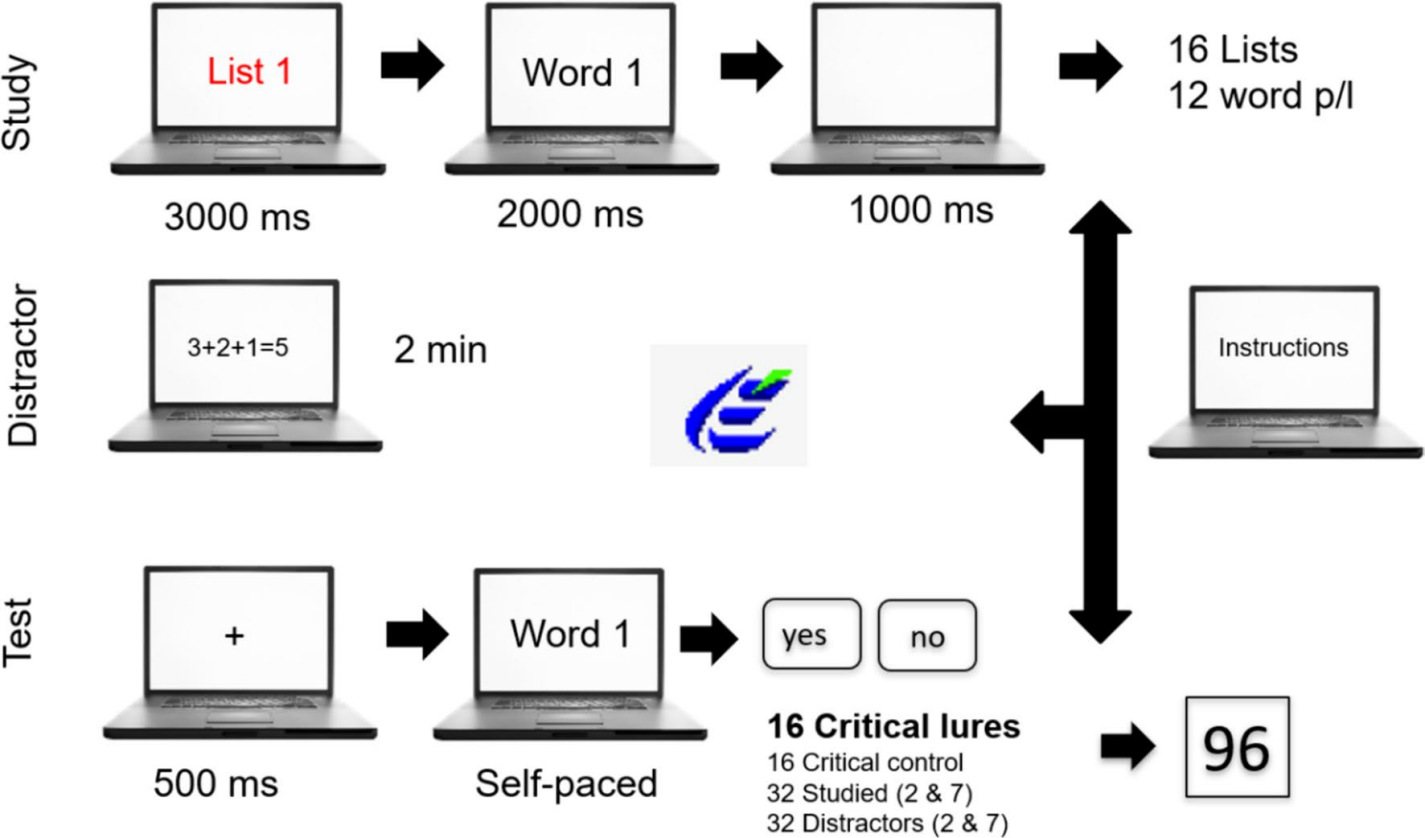
Experiment procedure

Figure 1 depicts a schematic view of the procedure.

## Results

All participants performed the distractor task correctly and no one got a score under 80%. The statistical analysis was performed only on participant’s “yes” responses in the recognition task.

### Correct recognition

To test correct recognition, a one-way ANOVA with repeated measures was performed comparing the different types of words: critical, critical control, distractor, and correct. Results showed that type of word was significant [*F* (23) = 138.89, *p* < 0.001]. Pairwise comparisons (Bonferroni) demonstrated that the proportion of “yes” responses for “studied” items (*M* = 0.75; *SD* = 0.07) was higher than for both “distractors” (*M* = 0.15; *SD* = 0.011) and “critical control” (*M* = 0.26; *SD* = 0.16) words (all *ps* < 0.0001). No differences were found between correct and false recognition (*p* = 0.44).

### False recognition

**Fig. 2:**
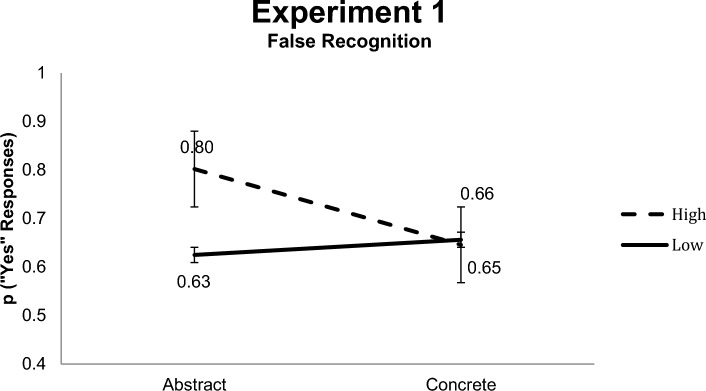
False memory effect in experiment 1

The mean proportion for “yes”-responses to critical lures was 0.68 (*SD* = 0.15). To test the relationship between concreteness and this false recognition effect (that was as high as the correct recognition), an ANOVA with repeated measures was performed. Results revealed a statistically significant interaction [*F* (1,23) = 4.96; *p* < 0.05] between concreteness and BAS, indicating that false recognition of critical words was higher for abstract words in the high BAS condition (*M* = 0.80; *SD* = 0.24) than for abstract critical words with low BAS (*M* = 0.63; *SD* = 0.29), as well as for weakly associated concrete words (M = 0.66; SD = 0.23) and concrete words with high BAS (M = 0.65; SD = 0.30) (see Fig. 2). No other effect reached statistical significance.

### Discussion

The main goal of this experiment was to explore the impact of the relationship between word concreteness of the critical lure and the backward association strength between this word and the list of its associates over false recognition. Interestingly, we found that false recognition was higher for those abstract lures that were strongly associated with their list. These results are in concordance with Crutch (2006) and Duñabeitia et al. (2009) and are in favor of QDR. From the psycholinguistic perspective, these results are also consistent with the idea that abstract and concrete concepts have a difference in processing, leading concrete words to be more accurate and faster to be processed. This is, a high number of false memories (in the case of abstract words), could be showing a less effective editing process to filter them out. Thus, the association will be the mechanism that participants use to accept an abstract not presented word as presented.

Since association has been describing in priming experiments as a short phenomenon, it would be interesting to assess the encoding phase to explore the role of this variable on false recognition. An electrophysiological approach would suit perfectly to do so, since this technique emphasizes the time curse of the neural activation that underlies the behavioral/cognitive processing regarding both to concreteness representations and to the creation of false memories. That is the goal of the next experiment.

### Experiment two

The objective of this experiment was to examine the neural correlates of false memories and their relationship with the behavioral results found in earlier experiments. The concreteness exploration in the brain has produced clear results about the neural mechanisms that underlay this difference. fMRI studies, for example, have shown that there seem to be neural correlates that differ from abstract and concrete concepts. Specifically, Binder et al. (2005) have found that there were areas in the left lateral temporal lobe that were equally activated by both concept types, whereas bilateral regions including the angular gyrus and the dorsal prefrontal cortex were more engaged by concrete words. In the field of the temporal domain, EEG has been used to test the time curse of abstract and concrete activation. Different authors have found that event-related brain potentials (ERPs) for concrete words usually show a long-lasting negativity (also called N700) compared to ERPs for abstract words (Barber et al.^2013^; Holcomb et al. 1999; Huang et al. 2010; Kanske and Kotz 2007; Kellenbach et al. 2002; Kounios 2007; West and Holcomb 2000). However, there have been also results that have found an earlier wave, peaking around 400 ms that is different for both abstract and concrete words. This N400 effect is crucial since this time window has been frequently associated with semantic processing and candidate activation (Rabovsky et al. 2018, 2012; Rabovsky and McRae 2014). The main goal of this experiment was to unravel the neural effect of studying word lists associated with abstract and concrete words.

## Method

### Participants

A total of 24 psychology students (8 men) took part in this experiment. The mean age was 20.50 (*SD* = 2.12) and all of them had normal or correct-to-normal vision. Participants were informed about the experiment’s characteristics and were asked to sign a consent form.

### Materials

Same as exp. 1

### Procedure

Before the start of the experiment, participants were seated in a quiet room and then they were fitted with an EEG recording cap. After that, participants were instructed to memorize sixteen twelve-word lists for an upcoming memory test. Each list was presented word by word and each word was presented for 2 s. Right from the beginning, the list label was presented for 5 s. The test phase was immediately presented after participants finished a 2-min interference task. Each trial started with a fixation cross that remained on the screen for 1 s, followed by a 500 ms blank screen and the memory probe, that lasted 1000 ms Subsequently, a blank screen was presented for 2 s, and later participants were asked to give a “yes/no” response to the memory probe with no time pressure imposed.

### EEG recording and processing

Scalp voltages were collected from a 60 Ag/AgCl electrode setting, which were mounted on an elastic cap (*ElectroCap International, Eaton USA 20–10 system*) referenced to the earlobes. Four additional electrodes were used to monitor eye movements and blinking. Inter-electrode impedances were kept below 10 KΩ. EEG was filtered at 120 Hz and digitalized at a sampling rate of 500 Hz.

At the end of the data collection, the EEG was transformed to average reference, and later eye movements and blinks were corrected using an ocular correction method (Gratton and Coles 1983). Afterwards, the EEG was transformed to the mean voltage reference and then the eye movements and blinks were corrected using the ICA (Principal component analysis) eye correction method. This method was chosen because of the advantage of removing ocular artifacts from this decomposition for signal cleaning (see Jung et al. 2000 for a review on the subject). A 0.032 Hz low-pass filter was then applied, along with a 30 Hz high-pass filter. The EEG was then segmented, creating windows between 200 ms prior to stimulus presentation and 1700 ms after stimulus presentation. Baseline correction was performed from 200 ms pre-stimulus. The rejection of artifacts was carried out semi-automatically, allowing a maximum voltage of 50 μV/ms. After averaging by condition, a measure of Global field power (GFP) was obtained, which allows solving possible problems related to the reference processes, in addition to a few other benefits about the use of this method (Murray et al. 2008). Finally, the overall average (or Grand Average) was performed on the GFP under all experimental conditions, both in the coding phase and in the test phase. The statistical analysis was performed creating regions based on laterality (left/right hemisphere) and placement (anterior, central, posterior).

## Results

### Behavioral results

**Fig. 3:**
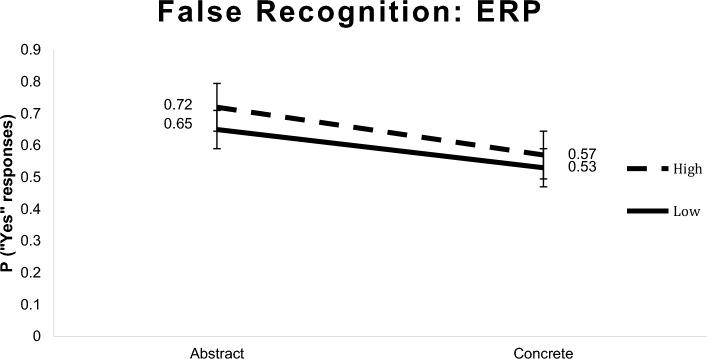
False memory effect: ERP

An ANOVA with repeated measures comparing concreteness (abstract vs. concrete) and BAS (high and low) showed that the abstract critical words generated higher rates of false recognition in comparison with the concrete ones [*F* (1,22) = 6.58; *p* < 0.05]. The *F* test performed contrasted the simple multivariate effect of concretion in each BAS level and reflected that the proportion of “yes” answers given to high BAS critical words was higher compared to those given to abstract critical words of low BAS [*F* (1,22) = 7.30; *p* < 0.05] (see Fig. 3).

### Encoding phase

Segments recorded in the encoding phase were sorted out according to participants’ latter elicitation/not elicitation of the critical lure. Global field power was used to average across all the electrodes. One participant was removed from the final analysis due to the lack of valid segments in one of the 2 experimental conditions due to the number of artifacts in the relevant segments. Since there were less than 2 segments per condition, this is unsuitable for exploring the variance. Because different lists induced memory illusions at different rates across participants (i.e., false alarm rates were not equal across the participants and so the valid segments selected from the encoding phase was not the same for all participant), we decided to remove randomly segments in one of the conditions, so they were paralleled.

### N400

**Fig. 4:**
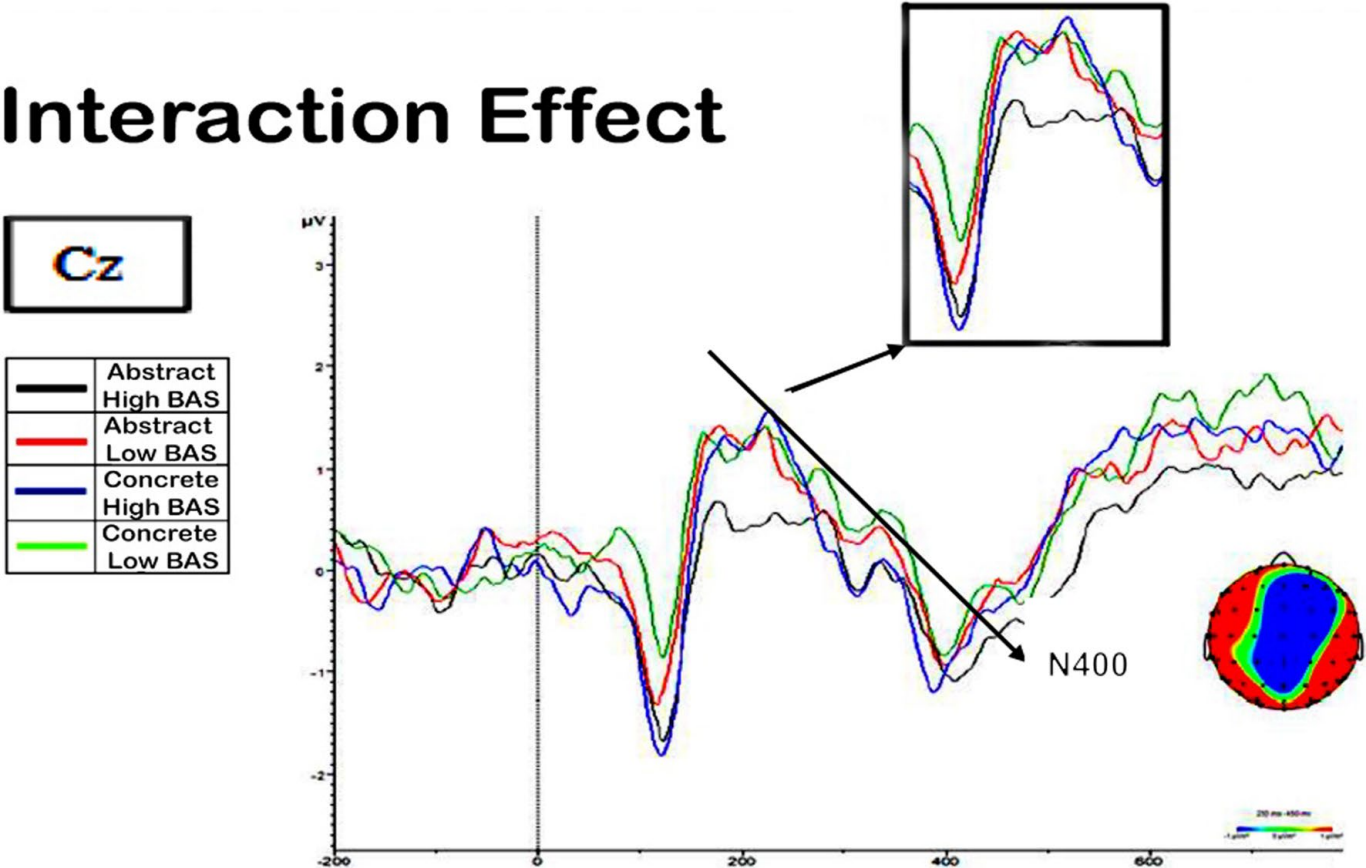
False memory effect: N400

An ANOVA, with elicitation (elicit vs no-elicit), concreteness (concrete vs abstract) and BAS (high vs low) in the activity obtained in Cz electrode and in the time-window 250–350 ms showed a triple interaction, statistically significant among elicitation, concreteness, and BAS [*F* (2,10) = 4.63; *p* < 0.05), reflecting those words highly associated to abstract lures showed a reduced N400 amplitude in comparison with the rest conditions but only in the elicitation condition (See Fig. 4.In the figure, the effect is highlighted so the wave that represents the abstract highly associated words is showing a reduced n400 effect.

## Discussion

The main goal of this experiment was to explore the neural mechanisms of false recognition in both abstract and concrete words, by manipulating the most relevant variables that affect it: concreteness and association strength. Specifically, we focus on the encoding test, and we created a condition where we could compare both eliciting and not eliciting false memories. The idea behind this approach was that differences may arise when studying lists that will produce a false memory. We remark on three important issues in this experiment. First, we found an early N400 concreteness effect, which we can interpret as an effect of the differential associative activation. Indeed, the N400 component has been considered a neural marker of semantic processing. It is often induced by words or sensations that are incongruent or semantically unexpected within a specific context. While the N400 is not directly associated with false memories, it has been researched in concreteness and semantic processing, which may indirectly relate to false memory generation. The fact that it appears so early indicates that association should be playing an important role in creating the representation of the critical word (or false alarm).

In fact, research has demonstrated that concrete words often evoke smaller or less negative N400 amplitudes compared to abstract words. This trend shows that concrete words benefit from facilitated semantic processing compared to abstract words (Kutas and Federmeier 2011; West and Holcomb 2000). Although our data show a decreased N400 effect for the abstract high associated lists, this effect is not exactly a concreteness effect since it comes from concrete and abstract words that are associated with a critical not presented word. One way to interpret these contrary results is in line with the Associative Activation theory, which stablishes that association is an important organizational principle for abstract words. This early N400 effect can be showing that at encoding, associative activation could be used to enrich the semantic processing that will lead to a false recognition just for the abstract words.

### General discussion

Our main goal in this study was to evaluate the role of word concreteness on false memories by using the DRM paradigm, which allows us to do so in a straightforward and controlled way. In the first place, we ran a standard experiment to explore roughly the interaction between association strength (BAS) and concreteness. Results showed that abstract words generated more false recognition, especially if BAS is high. This result fits perfectly with the QDR theory (Crutch and Warrington 2005) because the association is crucial for processing abstract but not for processing concrete concepts. Secondly, we sought to replicate the behavioral findings with a electrophysiological approach, so we could gather convergence evidence to our claim of the importance of association and concreteness in creating false alarms. Both experimental approaches allowed us to explore the organizational principles of semantic processing from an automatic, yet controlled way.

Regarding association, it is important to clarify that in QDR theory, association refers to the link that exists between two concepts that are either similar in meaning or tend to co-occur in language. These concepts are stored in semantic memory and depending on the nature of the link, they can form a qualitatively different semantic network. Thus, based on theories of spreading activation, the processing of a concept activates not only its representation but also the representation of related concepts (Collins and Loftus 1975). Theories explaining the formation of false memories often relies on this model to explain why a non-presented word is included in the set of “yes”-responses when participants are asked to decide if a memory probe has been studied or not. Specifically, the Associative Activation Theory proposes that a lure is activated due to the processing of a list of the lure's associates.

Regarding the data collected in this study, it seems probable that activation that occurred at the encoding phase, just at the moment where the critical word is receiving activation from its associates. As the association is crucial for the first ones, there may have also been a summation of activation both at study and test that caused the effects we found in both experiments in favor of abstract words, just as Huff and cols (2020) have recently suggested. The ERP study seems to confirm this, as the study phase was explored subsequently. Firstly, behavioral data was in line with our previous experiments. Secondly, when participants were studying word lists that eventually generated a false alarm, but only the abstract condition showed more amplitude in the N400 peak than the other conditions. Those results can be seen as a reflection of a primary activation process for abstract words, which mainly rely on association strength.

Taken together, we propose a DIM-HA effect (Differential illusion memory for high-associated abstract concepts), which is an interesting theoretical approach, as it provides another way to explore the concreteness effect from the false memory perspective. It constitutes the first time where false alarms explored the concreteness effect with the EEG technique. Further research needs to be done on this topic to delve deeper into this phenomenon, specially regarding to the duration and the propagation of the active information.

In sum, this study provides convergent evidence that contributes to advance in the knowledge of the possible mechanisms that lead to the creation of false recognition, and, at the same time, adds useful evidence for the concreteness debate. The interaction between association and concreteness both from the psycholinguistic and from the memory fields will build a stronger case for the explanation of the cognitive domains of language and memory regarding the representation of concrete and abstract concepts.
